# Recalibrating the anti-corruption, transparency, and accountability formula to advance public health

**DOI:** 10.1080/16549716.2019.1701327

**Published:** 2020-03-20

**Authors:** Aneta Wierzynska, Sarah Steingrüber, Roxanne Oroxom, Sebastian Bauhoff

**Affiliations:** a Ethics Office, The Global Fund to Fight AIDS, Tuberculosis and Malaria, Geneva, Switzerland; b Independent Global Health Expert, Berlin, Germany; c McCourt School of Public Policy, Georgetown University, Washington, DC, USA; d Department of Global Health and Population, Harvard T.H. Chan School of Public Health, Harvard University, Boston, MA, USA; e Center for Global Development, Washington, DC, USA

**Keywords:** Anti-Corruption, Transparency and Accountability, Anti-corruption, risk assessment, global health, fraud, risk

## Abstract

Policy-makers, implementing organizations, and funders of global health programs aim to improve health care services and health outcomes through specific projects or systemic change. To mitigate the risk of corruption and its harmful effects on those initiatives, health programs often use multiple anti-corruption mechanisms, including codes of conduct, documentation and reporting requirements, and trainings. Unfortunately, the introduction of anti-corruption mechanisms tends to occur without an explicit consideration of how each mechanism will affect health services and health outcomes. This may overlook potentially more effective approaches. In addition, it may result in the introduction of too many controls (thereby stymying service delivery) and a focus on financial or procurement-related issues (at the expense of service delivery objectives). We argue that anti-corruption efforts in health programs can be more effective if they prioritize addressing issues according to their likelihood and level of harm to key program objectives. Recalibrating the anti-corruption formula in this way will require: (i) extending responsibility and ownership over anti-corruption from subject experts to public health and health system specialists, and (ii) enabling those specialists to apply the Fraud Risk Assessment methodology to develop tailored anti-corruption mechanisms. We fill a documented gap in guidance on how to develop anti-corruption mechanisms by walking through the seven analytical steps of the Fraud Risk Assessment methodology as applicable to health programs. We then outline best practices for any anti-corruption mechanism, including a focus on quality health delivery; the alignment of actors’ incentives around the advancement of health objectives; and being minimally corruptible by design.

## Background

As health ministries, implementing organizations, and global health funders work toward universal health coverage (UHC), they are increasingly realizing the need to effectively and sustainably prevent, deter, and detect corruption. To that end, the World Health Organization (WHO), United Nations Development Programme (UNDP), and Global Fund launched the Alliance for Anti-Corruption, Transparency, and Accountability in Health (the Alliance) in 2019 to initiate a new dialogue around current challenges and potential solutions.

At two events organized by the Alliance, participants agreed that current corruption prevention models are not entirely fit for purpose. Indeed, there is limited evidence that ‘traditional’ anti-corruption mechanisms (e.g. integrity policies and codes of conduct) meaningfully prevent corruption [1]. In fact, poorly designed anti-corruption mechanisms may paradoxically generate corruption risks, e.g. through increased complexity and rigidity of financial and procurement processes. In this way, they may also stymie the effective delivery of the health programs they seek to protect [2,3]. The persistent challenge in identifying effective and sustainable methods of corruption prevention, deterrence and detection is a consequence of the fundamental limitations of the traditional approach to anti-corruption.

Insights emerging from the Alliance highlight that it is possible to design tailored anti-corruption models. This aligns with the recommendations of globally recognized audit, internal control, fraud examiner, and enterprise risk management standard setters that anti-corruption mechanisms should be carefully tailored to their context through the application of Fraud Risk Assessment (FRA) methodology. Despite this backing, the FRA methodology has been variably applied in the global health context. A review of health donors’ integrity systems found that ‘over half the agencies that require corruption risk assessments do not have detailed guidance on how they should be done or what should be included’ [4]. Additionally, the review found ‘the much more problematic level was determining how risk assessment[s] should affect the actual design of projects, and controls within the projects’ [5].

We argue that addressing corruption in the health sector requires recalibrating traditional anti-corruption mechanisms in two ways. First, by expanding traditional ownership over anti-corruption beyond financial and procurement experts to include public health and health systems specialists. Second, by enabling those specialists to apply the FRA methodology to craft customized mechanisms that mitigate the risk of corruption while affirmatively advancing public health objectives.

## The status quo and its limitations

‘Corruption’ is shorthand for any intentional abuse of power for private gain [6]. It follows that corruption can be performed by any actors within any process, including hiring/human resources procedures; supervision systems; monitoring & evaluation (M&E); and delivery of prevention, treatment, and support interventions. As the traditional anti-corruption approach focuses primarily on compliance within input-heavy procurement and financial processes, opportunities for abuse in other processes across the health systems may be insufficiently mitigated.

When relying on such input-heavy compliance processes, this also diverts focus and resources from ensuring quality delivery of health services [7], introduces operational inefficiencies, and generates low absorption of health resources [8,9]. In extreme cases, funding for health projects may be suspended because of corruption, ignoring the health improvements those projects may have achieved. Indeed, interrupting funding may threaten additional or past health gains [15].

Furthermore, corruption is always performed intentionally and knowingly, meaning the actors are aware that their behavior is non-compliant, regardless of what traditional policies, codes of conduct and anti-corruption trainings tell them. In fact, corruption is inherently hidden because actors take effort to conceal their abuse by generating the perception of compliance (e.g. procurement tender minutes, financial invoices, programmatic activity reports and data), and therefore a false sense of assurance.

## The paradigm shift

It is possible to generate an effective, sustainable anti-corruption model that prioritizes health objectives. This can be accomplished by applying the FRA methodology, an established method used to design context-specific corruption prevention controls, recommended by internal control, audit and fraud examiner associations [10,11]. The FRA is founded on a risk-based approach, which differs from the more traditional ‘compliance’ approach of anti-corruption mechanism design. The compliance approach is predicated on zero risk appetite, and therefore designs mechanisms to block, check, and document all incidents of fraud. The risk-based approach counsels designing controls to focus on those incidents that could most adversely affect process objectives. The compliance-based approach assumes that, as long as all the rules appear to be followed, and all the required documents and signatures are in place, this must mean that fraud and corruption did not occur. The risk-based approach poses a different question: Could managerial override, collusion, and the inherent forgeability of documents generate the perception of compliance and instead mask diversion, corruption, and ultimately non-delivery of health services? The risk-based approach recognizes the reality of fake-able compliance and documents, as well as the practicalities of limited resources, the need for cost-effectiveness, and the unintended consequences of excessive red tape. It counsels that preventive efforts should mitigate the greatest risks, rather than all risks, and in a manner that generates authentic assurance.

The FRA methodology can guide project and health system designers in tailoring anti-corruption mechanisms (e.g. controls, reporting requirements, incentives) within a process (e.g. data collection, intervention delivery, payroll) to optimally advance health outcomes and service delivery, while also preventing the most pernicious forms of corruption. FRAs can be applied before or during (ideally before) the design or implementation of any initiative. Be it a process, program, intervention or evaluation, the analytical steps remain the same:

### Bring together the right fiduciary and programmatic professionals

FRA exercises should be undertaken by all the experts required to operationalize the selected process, taking particular care to draw on the perspectives of both the programmatic and the fiduciary teams. An independent moderator with expertise in fraud risk assessment methodology should mediate the discussion.

### Define the programmatic objectives

In a health system, all processes exist because they directly or indirectly support delivery of health services/programmatic objectives. A risk-based approach proposes to evaluate whether and how processes within a health system contribute to the overall objective they ultimately serve, i.e. improving health outcomes. For example, a payment process is needed to enable a programmatic activity to be delivered on time, with good quality, and at a fair price. Secondary objectives, such as ensuring compliance with financial management protocols or preventing corruption, remain relevant but only as far as they facilitate the primary health objective.

### Incorporate all processes that are critical to achieve the programmatic objectives

When health services/programmatic objectives anchor the analysis, the scope of processes naturally expands from financial management and procurement to other processes required to deliver services and collect reliable data, such as planning, logistics and supervision. For example, achieving the objective to reduce HIV transmission requires ensuring not only that the correct antiretrovirals (ARVs) are procured at an appropriate price; but also, that facilities are adequately stocked; the health workforce is qualified; and the correct information is provided to patients and communities. Each of these sub-processes may be intentionally abused, therefore necessitating bespoke anti-corruption measures.

### Brainstorm forms of intentional abuse

Apply Fraud Triangle Theory to brainstorm the scale and nature of intentional abuse. This theory suggests that fraud risk increases as opportunities for abuse increase (e.g. it’s easy; no one will know), as the incentives or pressures to engage in abuse increase (e.g. I need the money, I need to pay to keep my job), and as rationalizations materialize (e.g. everyone is doing it) [12]. Here, it helps to map processes in order to identify institutional roles within and around processes, and then evaluate the incentives and opportunities for abuse to which such roles are exposed. For example, health workers may have an incentive to exaggerate patient adherence to antiretroviral therapy if reported levels have financial or reputational implications. Contemplating possible incentives and opportunities will not only bring to light a universe of potential schemes; it will also identify the perverse incentives to target when designing anti-corruption mechanisms. Corruption typologies, such as that from Transparency International, can provide a systemic overview of typical corruption vulnerabilities affecting health systems [13].

### Rate the risk level of the corruption schemes according to which would have the most adverse effect on health objectives

Ranking risks in this way shifts focus away from small-scale corruption schemes that have little bearing on the health objectives and towards schemes that have a more harmful effect.

### Design customized mechanisms to prevent or deter selected schemes

Anti-corruption mechanisms should target the most harmful and likely schemes, as well as balance the benefits of risk mitigation against the costs associated with deploying the mechanism – always against the underlying health objective. This requires considering: (i) the steps to the process; (ii) who performs which step of the process; (iii) how conditions of payments are structured; (iv) transparency requirements; (v) documentation requirements, (vi) the design of verification, supervision and oversight mechanisms, and (vii) incentives and sanctions for the various stakeholders involved in the process.

### Evaluate your anti-corruption mechanisms for ‘corruptibility’

Anti-corruption mechanisms should be sufficiently independent, difficult to manipulate or evade, generate no or few opportunities for abusing power, and be resilient to forgery. For example, the most popular and default anti-corruption mechanism – a documentary record – is forgeable by its very nature. However, even independent supervision, verification, and evaluation activities can be corrupted if it is possible for corrupt actors to know in advance where independent parties go, what they see, and who they speak to. In many cases, slight adjustments to a mechanism’s design can increase its effectiveness, e.g. supervision visits can be unannounced.

Effective corruption prevention mechanisms will share common characteristics:

### They will focus on ensuring that the health/process objectives are achieved

For example, an objective of a supervisory visit to a training session is primarily to evaluate the quality of the training. The unannounced nature of the visit would also deter faking the training, but this is a secondary characteristic of the core control and its focus on the quality of the training. This is because the best practice in anti-corruption is often synonymous with best practices in objectives-focused process design and quality assurance.

### They will target actors’ incentives by aligning their interests to the health objective, thereby deterring abuse

For example, in a procurement process, a portion of the payment to a supplier can be conditioned on an independent verification that the supplier delivered in time and in quality. This will encourage the supplier to focus on the health objective – the delivery of the goods or services. At the same time, the risk of not being able to recuperate a large portion of the full contract amount will deter the supplier from diverting funds and underperforming for that reason.

## Conclusion

Tackling corruption is essential for improving the use of scarce resources, yet traditional anti-corruption mechanisms are often insufficiently fit-for-purpose. This can generate a false sense of security as well as ignore the new costs and opportunities for corruption these mechanisms may create. Despite their lack of demonstrable success, traditional and compliance-based anti-corruption mechanisms continue to be frequently used.

Reducing corruption in health systems requires a re-centering around the ultimate health objectives. That, in turn, requires shifting prevention models away from a ‘zero-tolerance’ mindset and towards a risk-based approach. The ACTA Alliance 2019 global consultation on this topic among corruption experts, auditors, health economists, and public health specialists highlighted that a risk-based approach may be the key to effectively, efficiently and sustainably improving corruption prevention [14]. Health actors should incorporate the FRA methodology into their control and assurance model design, test it, and publish learnings to advance best practice in this area.

## Paper context

Corruption harms health care systems, but commonly used anti-corruption controls, like financial accounting controls and compliance programs, are not optimally fit for purpose. This article outlines a methodology for tailoring corruption prevention mechanisms within the health context. It answers a practitioner demand for guidance on how to (1) more effectively prevent corruption in health (2) design corruption prevention mechanisms that accelerate rather than stymie health objectives and (3) operationalize a risk-based approach in this domain.
